# Assessing acuity in pediatric emergency department triage: performance of a large language model

**DOI:** 10.1038/s44401-026-00128-6

**Published:** 2026-08-05

**Authors:** Kush Narang, Newton Addo, Christopher Y. K. Williams, Maytal Firnberg, Jean Feng, Aaron Kornblith

**Affiliations:** 1 Department of Emergency Medicine, University of California, San Francisco, San Francisco, CA USA; 2 Bakar Computational Health Sciences Institute, University of California, San Francisco, San Francisco, CA USA; 3 Quality Health Inc., San Francisco, CA USA; 4 Department of Epidemiology and Biostatistics, University of California, San Francisco, San Francisco, CA USA

**Keywords:** Diseases, Health care, Medical research

## Abstract

Pediatric triage performance varies across emergency departments (ED), contributing to ongoing challenges in pediatric emergency care. There is growing interest in using large language models (LLMs) to support more consistent triage decision-making in children. We evaluated an LLM’s (GPT-5-mini) ability to identify the higher-acuity child from pairs of de-identified clinical notes. Across 228,104 pediatric ED visits, the LLM achieved an overall accuracy of 0.73 (95% CI, 0.73–0.74) in identifying the higher-acuity child, with accuracy improving as acuity differences between visits increased. The LLM was less likely to be correct when the higher-acuity child was older (odds ratio, 0.62, 95% CI, 0.61–0.63) and when the age difference between children was large (0.75, 95% CI, 0.70–0.79). The LLM showed moderate overall accuracy in assessing pediatric acuity and demonstrated a tendency to prioritize younger children, similar to human performance. These findings highlight the need for pediatric-specific LLM evaluation and optimization before clinical use.

## Introduction

Each year, more than 30 million children visit emergency departments (EDs) in the United States, and around 85% receive care without pediatric-specific specialists^1^. Children treated in general EDs experience higher rates of medical error, portending worse outcomes and the “pediatric readiness gap”^2–6^. ED triage often determines the clinical trajectory of a child’s visit^7^. Yet non-specialty EDs demonstrate inconsistency and experience-related biases in triaging children due to infrequent pediatric exposure and variable triage processes^8–10^.

Large language models (LLMs) may offer clinicians an augmented way to deliver more consistent and specialty-level emergency care^11–13^. Prior studies show that off-the-shelf LLMs can perform near expert-level on ED triage acuity tasks in adult patients^14,15^. However, recognizing the limited evaluation studies on pediatric ED triage and mixed results on other pediatric benchmarks, LLMs may lack the training needed to perform reliably on children^16–19^. Without child-specific validation, these tools may replicate, rather than correct, existing performance gaps.

We conducted a retrospective evaluation of 228,104 pediatric ED visits from two academic children’s hospitals to assess an LLM’s accuracy in determining pairwise triage acuity compared with a clinical reference standard. We hypothesized that the LLM would perform worse than pediatric experts and would demonstrate age-dependent variation, similar to human triage.

## Results

We identified 358,040 pediatric ED visits during the study period, of which 228,104 met inclusion criteria. These visits were assembled into 114,052 comparison pairs. Summary statistics are available in Table 1. The study cohort includes 53.9% male and 15.7% White children. Immediate and non-urgent represent the least frequent ESI assignments, comprising 2.3% (*n* = 5354) and 5.0% (*n* = 11,439) of visits, respectively. Urgent and less urgent are the most common assignments, accounting for 38.9% (*n* = 88,709) and 35.3% (*n* = 80,556) of visits, respectively.

**Table 1:** Demographic characteristics of the cohort

	Prevalence (*n*)
Race/ethnicity	
Asian	10.1% (22,938)
Black or African American	15.3% (34,878)
Latino	38.2% (87,220)
White	15.7% (35,899)
Other (includes Declined and Unknown)	20.7% (47,169)
Sex	
Male	53.9% (122,909)
Female	46.1% (105,112)
Other (includes Nonbinary and Unknown)	0.0364% (83)
Acuity level	
Immediate (ESI 1)	2.3% (5,354)
Emergent (ESI 2)	18.4% (42,046)
Urgent (ESI 3)	38.9% (88,709)
Less Urgent (ESI 4)	35.3% (80,556)
Non-Urgent (ESI 5)	5.00% (11,439)
Ages	
Infant (0 – <1)	14.4% (32,767)
Toddler (1 – <3)	20.8% (47,534)
Early childhood (3 – <6)	19.8% (45,154)
Middle childhood (6 – <12)	24.3% (55,509)
Adolescent (12 – <18)	20.7% (47,140)

This table presents characteristics for individual patients, rather than pair-level information. For all analyses, patient ages are grouped as specified in the table, in accordance with National Institute of Child Health and Human Development (NICHD) guidelines. Latino includes patients with a listed race of White or Other. A significant portion of the cohort’s race/ethnicity information is “Unknown” or “Declined”. The clinician-assigned ESI acuity distribution matches expectations for pediatric EDs at large tertiary care centers.

The LLM achieved an overall accuracy of 0.73 (95% CI, 0.73–0.74) in identifying the higher-acuity visit in a pair. Accuracy improved as the difference in ESI scores widened (Table 2). The LLM performed best on immediate-non-urgent pairs (0.96; 95% CI, 0.93-0.99), and worst on immediate-emergent pairs (0.61; 95% CI, 0.57–0.64).

**Table 2:** LLM accuracy, stratified by ESI scores

	Immediate	Emergent	Urgent	Less Urgent	Non-Urgent
Immediate	-	-	-	-	-
Emergent	0.61 (0.57–0.64)	-	-	-	-
Urgent	0.84 (0.83–0.86)	0.78 (0.77–0.78)	-	-	-
Less Urgent	0.92 (0.91–0.93)	0.88 (0.88–0.89)	0.65 (0.65–0.66)	-	-
Non-Urgent	0.96 (0.93–0.99)	0.94 (0.92–0.95)	0.81 (0.80–0.82)	0.70 (0.68–0.71)	-

The LLM also performed poorly on urgent-less urgent pairs (0.65; 95% CI, 0.65–0.66). These pairs were the most frequent, representing 50.1% of the study cohort. Removing urgent-less urgent pairs increased overall accuracy (0.78; 95% CI, 0.77–0.78). When we removed all one-level-difference pairs from analysis (immediate-emergent, emergent-urgent, urgent-less urgent, and less urgent-non-urgent), the overall LLM accuracy increased (0.87; 95% CI, 0.87–0.87).

### Age-dependent variation in LLM accuracy

**Fig. 1:**
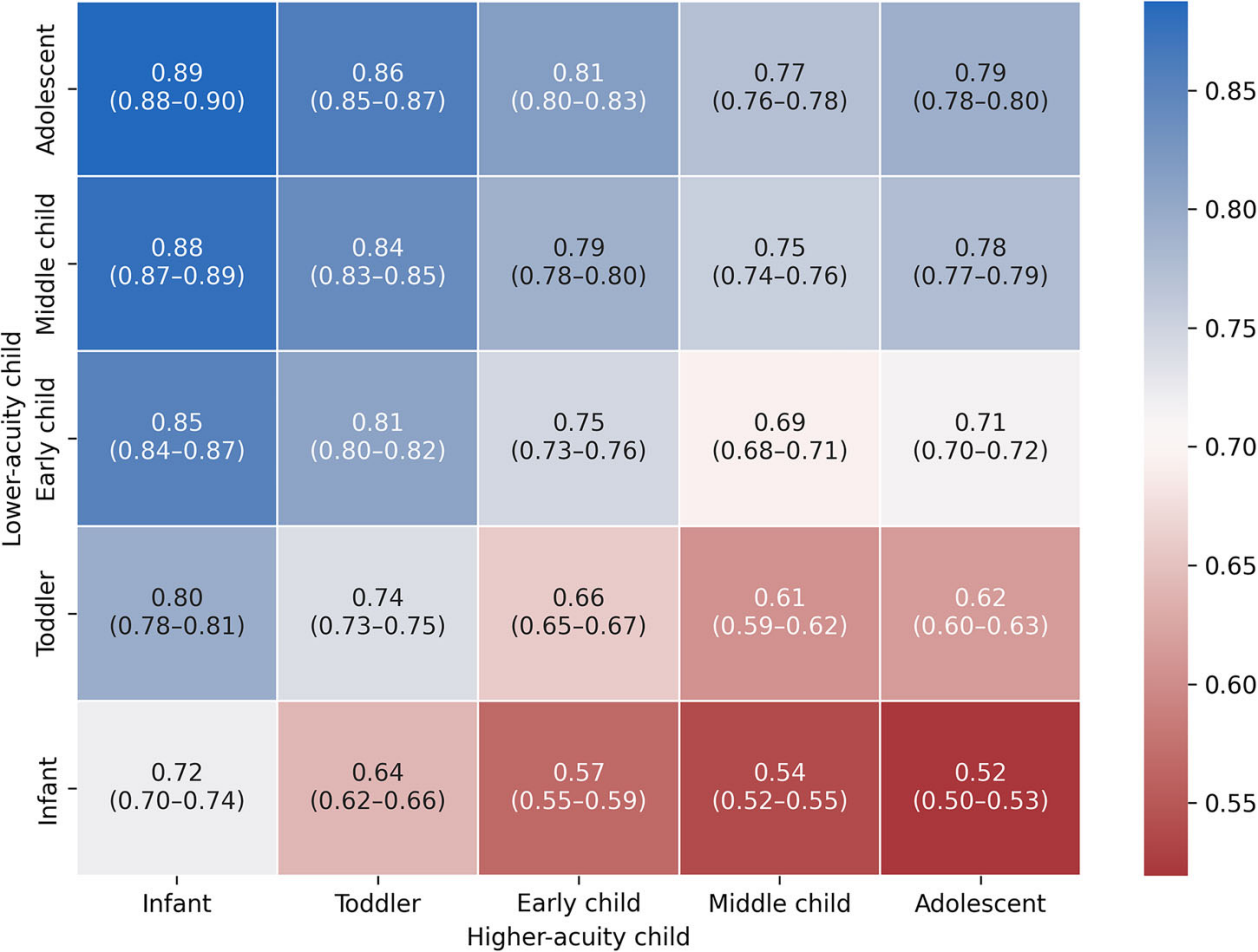
The LLM exhibits age-dependent variation in identifying the higher-acuity child in a pair. All one-level increases in the lower-acuity child’s age (e.g., steps up a column) are associated with a significant improvement in LLM accuracy, except for the step from middle child to adolescent in the infant column (diff. 0.01; 95% CI, –0.01–0.02). Similarly, all one-level increases in the higher-acuity child’s age (e.g., steps to the right across a row) are associated with a significant decrease in LLM accuracy (*p* < 0.05), except for the step from middle child to adolescent in the infant (–0.02; 95% CI, –0.04–0.004) and toddler rows (0.01; 95% CI, –0.01–0.03).

Figure 1 presents LLM accuracy, stratified by the ages of the higher-acuity and lower-acuity child, respectively. Along the diagonal, where children are in the same age group, accuracy remains consistent, ranging from 0.72 (95% CI, 0.70–0.74) for infants/toddlers to 0.79 (95% CI, 0.78–0.80) for adolescents. These values align with the overall accuracy of 0.73. However, when the two children differ in age groups, LLM predictions often vary significantly.

LLM accuracy was lowest when the higher-acuity child was an adolescent and the lower-acuity child was an infant (0.52; 95% CI, 0.50–0.53). LLM accuracy was highest for the inverse pairing, where the higher-acuity child was an infant and the lower-acuity child was an adolescent (0.89; 95% CI, 0.87–0.90). In Fig. 1, increased LLM accuracy was associated with the lower-acuity child being older and the higher-acuity child being younger (moving up a column and left across a row, respectively). For each row, holding the age of the lower-acuity child constant, increases in the age of the higher-acuity child were associated with decreased LLM accuracy (*p* < 0.001).

The odds ratios for correct LLM predictions decreased when the two children differed by three age categories (odds ratio, 0.89; 95% CI, 0.85–0.94) or four age categories (0.75; 95% CI, 0.70–0.79), compared to same-age pairs (Table 3). The odds ratio for correct LLM predictions also decreased when the older child was higher acuity (0.62; 95% CI, 0.61–0.63). Age-stratified odds ratios for incorrect LLM predictions, adjusted for sex, race/ethnicity, age, and acuity differences between children, show similar patterns (eFig. S2).

**Fig. 2:**
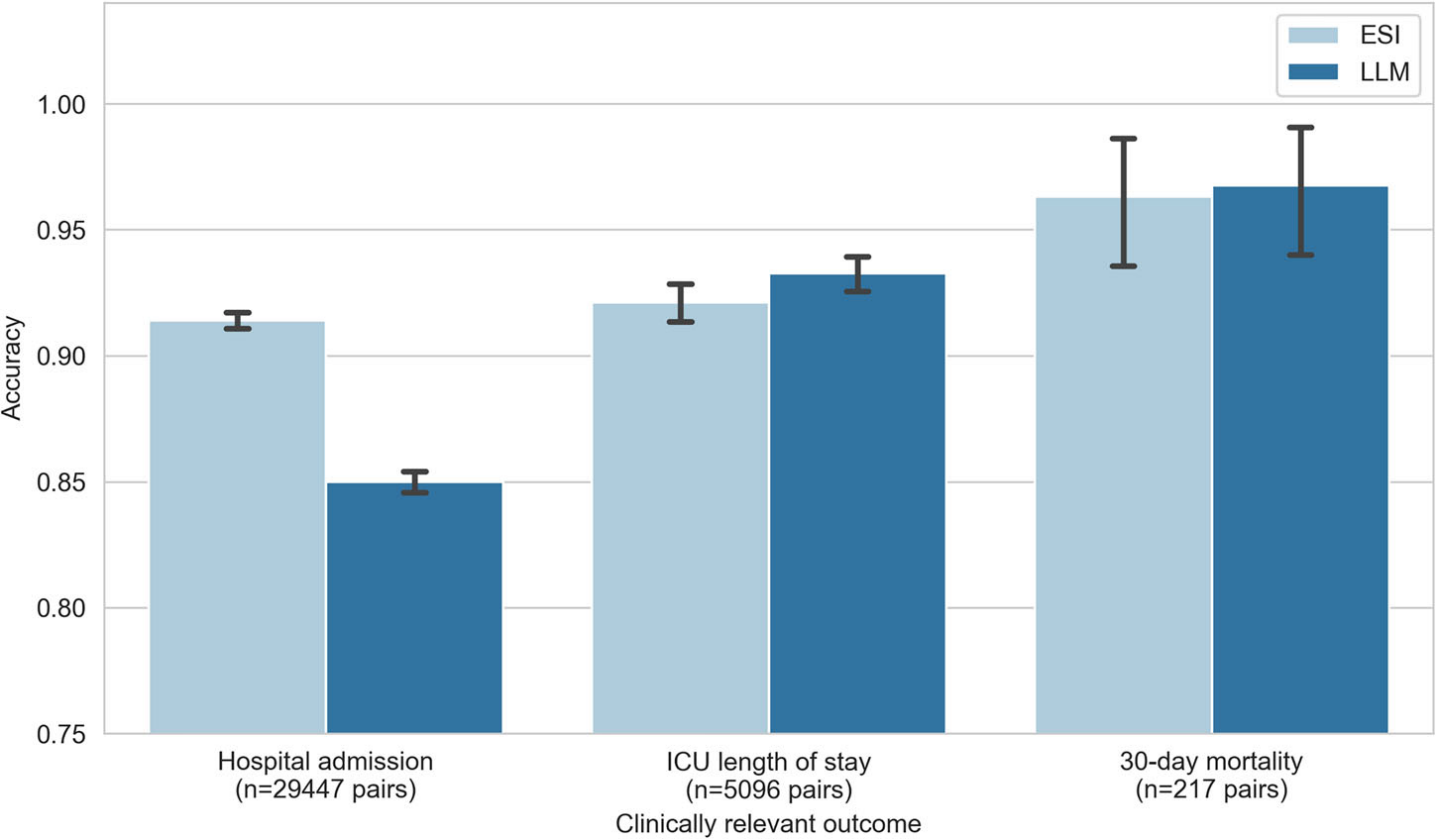
The LLM identifies 30-day mortality and greater ICU length of stay as well as ESI but struggles on hospital admission.

**Table 3:** Odds ratios for correct LLM predictions

	Odds ratio for correct LLM predictions (95% CI)
No age difference (same age group)	REF
Age difference, 1 group	1.05 (1.01–1.09)
Age difference, 2 groups	0.98 (0.94–1.02)
Age difference, 3 groups	0.89 (0.85–0.94)
Age difference, 4 groups	0.75 (0.7–0.79)
Acuity direction (+1 older, 0 same, −1 younger)	0.62 (0.61–0.63)

Age difference was measured as the number of age groups by which the two children differed, categorized as in Methods (infant, toddler, early childhood, middle childhood, adolescent). Acuity direction compares the age (in years) of the higher-acuity child to the lower-acuity child, encoded as +1 when the higher-acuity child is older, -1 when younger, and 0 when same age as the other child.

### Predicting clinically relevant outcomes

For the secondary objectives, we evaluated LLM performance in three outcome-stratified subsets (Fig. 2): hospital admission vs discharge (*n* = 29,447 pairs), greater ICU LOS vs shorter/no LOS (*n* = 5096 pairs), and 30-day mortality vs. 30-day survival (*n* = 217 pairs). Across all secondary outcomes, clinician-assigned ESI scores achieved predictive accuracies of 0.91 or higher. For hospital admission, ESI achieved a higher accuracy of 0.91 (95% CI, 0.91–0.92), compared to 0.85 (95% CI, 0.85–0.85) for the LLM predictions, a significant difference (*p* < 0.001). The LLM performed at least as well as the original ESI scores in predicting ICU LOS and 30-day mortality (*p* < 0.05).

## Discussion

Our study provides a large retrospective evaluation of an LLM’s accuracy in determining pediatric acuity using de-identified ED notes. The LLM demonstrated moderate overall accuracy and a consistent age-dependent tendency to prioritize younger children. The clearest example of this prioritization was among the pairings of infants and adolescents. When the adolescent was higher acuity, the LLM correctly identified the adolescent only half of the time. Conversely, when the infant was higher acuity, the LLM’s accuracy rose to nearly 90%, suggesting an underlying tendency to favor younger children as higher acuity. The rate of this younger-child prioritization also appeared to increase as the age difference between the children grew. This preference persisted across multiple age-stratified categories and may indicate a systemic tendency for the LLM to prioritize younger children, regardless of their true acuity level.

Our findings parallel well-described patterns in ED triage by clinicians, where younger children are often over-triaged and older children under-triaged^8,9^. Prior work also shows that pediatric ESI distributions are skewed toward additional urgent and less-urgent assignments^8,9^, a difference from adult populations that may contribute to LLM errors. Although we anticipated that LLM trends would mirror known biases in clinician-assigned ESI scores^8,9^, the high degree of age-dependent variation observed in this study highlights the unique challenges in applying off-the-shelf LLMs to pediatric triage. These LLMs are trained on all available data and are likely to receive significantly fewer training examples and less information on pediatrics, a potential cause of the decreased performance.

Rather than directly assigning ESI, our study investigated pairwise acuity determination, a more clinically useful task, for four reasons. First, the fundamental goal of triage is to determine a patient’s relative acuity ranking compared to others, rather than assigning a score. Triage systems like ESI are proxies that group patients by acuity, because individualized acuity ranking by clinicians is not practical; as an example, in ESI systems, most patients are assigned to the same two acuity groups (ESI 3 and ESI 4)^8,9^. However, because LLMs can efficiently process and rank information, they are better suited to individual risk assessment and relative comparison. Second, relative acuity assessment enables continuous patient monitoring. Rather than assigning a one-time ESI score, an LLM running in the background of a busy waiting room could continually update and adjust patient priority rankings, adapting to changing acuity and dynamic vital signs. Third, we designed this study to create a pediatric analysis complementing the well-established adult triage benchmark in Williams et al.^14^. Fourth, an additional aim of this study was to evaluate age-dependent variation in LLM acuity determination, rather than evaluating LLMs as deployment-ready solutions in real-world settings. The pairwise comparison task is better suited for identifying these relative variations, a key challenge in pediatric acute care.

Importantly, the LLM’s performance was lower than that reported in adult triage tasks using similar methodologies, despite our use of more advanced models and prompting techniques^14^. These differences align with the broader clinical understanding that pediatric emergency care requires distinct diagnostic approaches and training compared with adult care. The LLM was also less accurate than ESI in identifying hospital admission, even though performance was similar for ICU admission and mortality. Admission decisions often reflect hospital-specific practices rather than initial clinical acuity, which may limit how well a general-purpose LLM can predict this outcome.

Our results suggest that the best initial application of LLMs in ED triage could be in continuous patient assessment. In a busy ED waiting room, an LLM could act as an additional layer of safety by flagging dynamic changes in patient prioritization. The LLM would provide another data point for clinical teams, thereby augmenting clinician judgment, rather than replacing it. The age-dependent variation described in this study highlights the need for prospective multicenter studies and robust regulatory evaluations before deploying LLMs in place of humans in triage settings.

This study has several limitations. First, ED provider notes are written at a later time in a patient’s ED course than the initial time of ESI triage. During this time gap, a patient’s acuity may change, and the provider may document information that differs from what the initial triage team observed. To address this, we extracted only specific sections from the note, an approach used to approximate triage details in prior LLM studies^14^. Second, ESI is an imperfect reference standard and varies with provider experience and patient age^8,9^. We addressed this by including only visits from two high-volume children’s hospitals and demonstrating that our clinician-assigned ESI scores closely reflected clinically relevant secondary outcomes, including ICU admission and 30-day mortality. Previous studies have also used hospital admission as a reference standard for ESI scores^10^. Despite these mitigations, previous work has also shown that clinician-assigned ESI tends towards over-triaging younger children and under-triaging older children^8,9^, introducing potential bias in our reference standard. However, the ESI variation in favor of younger children is in the same direction as the pattern shown by the LLM. This knowledge suggests that, even when compared to a reference standard that already favors younger children, LLM predictions still show a further preference towards younger children. Therefore, using clinician-assigned ESI as a reference standard should not change the overall conclusions on age-dependent variation, and if anything, may underplay the degree of the LLM’s preference for younger children. Third, this study evaluated a single LLM with a specific prompt. Improved LLMs and new prompting strategies would likely result in different performance characteristics. Fourth, some notes used for model selection and prompt optimization were also included in the evaluation cohort. This overlap represented less than 1% of the dataset and likely had minimal impact on overall performance.

Our findings emphasize the need to independently verify LLM performance in children, regardless of successes reported in adult populations. Achieving reliable performance in children will likely require models optimized for pediatric-specific clinical cases, guidelines, and datasets. Additional large-scale studies evaluating LLMs on key pediatric tasks will be essential to identify performance gaps, inform model refinement, and guide the development of safer and more equitable tools for acute care in all children.

## Methods

### Study design and study participants

Our study is a retrospective evaluation of all pediatric ED visits at two tertiary-care academic children’s hospitals in Northern California between February 2014 to January 2025. Both EDs use the five-level Emergency Severity Index (ESI) system for patient triage: immediate (most acute), emergent, urgent, less urgent, and non-urgent (least acute)^20^. All data were accessed through the University of California, San Francisco (UCSF) Health de-identified clinical data warehouse and were exempt from Institutional Review Board review and informed consent (UCSF HRPP). This study followed the TRIPOD-LLM reporting guidelines^21^.

### Cohort assembly and data preparation

We included all ED visits where the child was younger than 18 years old and had a documented ESI. We excluded visits with missing or incomplete ED clinician notes. To replicate triage, we followed prior published approaches and selected the first ED clinician note from each visit, analyzing only four sections: chief complaint, history of present illness, review of systems, and triage vital signs^14^. Age was categorized into: infant (0 years old [yo] to <1 yo), toddler (1 to <3 yo), early childhood (3 to <6 yo), middle childhood (6 to <12 yo), and adolescent (12 to <18 yo)^22^. The full cohort selection flowchart and note preparation strategy are available in eFigure 1.

### Outcomes

The primary outcome was the LLM’s accuracy in identifying the higher-acuity child, defined by clinician-assigned ESI scores, and the LLM’s age-dependent variation across pediatric age groups. Secondary outcomes included the LLM’s accuracy in identifying the child with subsequent hospital admission, greater intensive care unit (ICU) length of stay (LOS), and 30-day mortality.

### LLM inference

We followed the pairwise comparison approach described by Williams et al. with minor modifications^14^. To reflect the natural distribution of triage acuity in the pediatric ED, we randomly paired pediatric visits with different ESI scores. Visits were randomly sampled without replacement and placed into pairs of children with two different ESI scores. Each child is represented in only one pair, and pairs were assembled until any of the five ESI categories ran out of eligible children. Children in each pair were randomly assigned to being first (Patient A) or second (Patient B) in the prompt to control for any influence caused by their order. The LLM reviewed each pair and indicated which child was of higher acuity.

We performed prompt engineering and model selection on a 1000-note subset of the overall cohort (eTable). We selected OpenAI’s GPT-5-mini model (ID: gpt-5-mini-2025-08-07; medium reasoning) using a prompt that includes guidelines from the ESI Handbook for all subsequent analyses (ESI-inspired prompt; eData). We performed all inference on the HIPAA-compliant UCSF Secure Azure OpenAI environment. Detailed prompts and parameters are provided in Supplement 1.

### Data analysis

In our primary analysis, clinician-assigned ESI scores served as the reference standard for patient acuity. In the secondary analysis, the reference standard for patient acuity was redefined to one of three clinically relevant outcomes: hospital admission, ICU LOS, and 30-day mortality. In the hospital admission subset, we defined the admitted child as higher acuity and the discharged child as lower acuity. In the ICU subset, we defined the child with the longer ICU LOS as higher acuity and the child with the shorter LOS or no ICU admission as lower acuity. In the 30-day mortality subset, we defined the child that died within 30 days as higher acuity and the child who survived at least 30 days as lower acuity. Each subset includes the pairs where only one child experienced the higher-acuity outcome.

We reported all accuracies with bootstrapped 95% confidence intervals (CIs) and determined statistically significant differences with a two-proportion z-test. We used the Cochran-Armitage test to identify statistically significant trends between accuracy and increasing ages of the higher-acuity child. All analyses were performed with Python 3.12 (Python Software Foundation).

### Ethics Approval

The UCSF Institutional Review Board (IRB; UCSF HRPP) has determined that studies using these de-identified data are exempt from IRB review and informed consent.

## Supplementary information

Supplementary materials

## Data Availability

The University of California, San Francisco does not permit the public release of the de-identified clinical notes used in this study.
