# Supplementary material for: Assessing acuity in pediatric emergency department triage: performance of a large language model
Source: Npj Health Syst. 2026 Aug 5;3:68. doi: 10.1038/s44401-026-00128-6 (PMC13438747; doi:10.1038/s44401-026-00128-6)
Supplement: Supplementary file 1 [file 44401_2026_128_MOESM1_ESM.docx]

**Online-Only Supplement: Assigning Acuity in Pediatric Emergency Department Triage: Performance of a Large Language Model**

**eFigure 1.** Cohort inclusion and exclusion flowchart.

**eTable.** Model selection and prompt optimization.

**eData.** The final optimized prompt provides Emergency Severity Index guidelines to the LLM.

**eFigure 2.** Adjusted odds ratios for incorrect LLM predictions demonstrate age-dependent trends in acuity determination.

**eFigure 1: Cohort inclusion and exclusion flowchart.**

ED visits with the following dispositions were excluded from the cohort, since these visits’ clinical notes may not contain enough information for fair LLM predictions: "Dismissed - Never Arrived", "LWBS before Triage", "LWBS after Triage", "Eloped", "AMA", "Dismissed - Diverted Elsewhere", "Send to L&D", "Observation", and "Dismiss - Lab Only (Non OED)" (collected from structured data). All note validation was performed programmatically in Python 3.12 (Python Software Foundation). All notes required a documented chief complaint or history of present illness. The review of systems and triage vital signs sections were optional but included if present. To best approximate the information known at the point of triage, we were intentional to never select the physical exam, medical decision-making, and other subsequent sections of the note. Manual review of three hundred notes confirmed that the desired sections were extracted accurately.

**eTable: Model selection and prompt optimization.**

We compared two prompting strategies. First, a “Basic” prompt was derived from the study by Williams et al. ([2024](https://jamanetwork.com/journals/jamanetworkopen/fullarticle/2818387)) examining LLM triage of adults, modified to incorporate the age of each child, a variable especially important in pediatric triage. Second, we created an “ESI-inspired” prompt (**Supplementary Data 1**), which incorporates guidelines from the Emergency Severity Index Handbook.

These strategies were evaluated in a 1,000-note subset of the overall cohort, balanced to contain 50 pairs of all ten ESI combinations (e.g., 50 pairs of ESI 1-2, 50 pairs of ESI 2-3, etc.). The unweighted accuracy provides the direct performance on these 500 pairs, while the weighted accuracy adjusts the result to reflect the natural ESI distribution in the larger cohort (e.g., few 1s and many 3s).

Due to similar weighted performance between gpt-5 and gpt-5-mini, we went forward with gpt-5-mini (medium reasoning) for all subsequent analyses, because it was approximately 5 times cheaper at the time of this study. This lower cost enabled a significantly larger final cohort than what would have been possible with the full gpt-5 model.

| **Model** *(medium reasoning)* | **Prompt** | **Weighted accuracy** | **Unweighted accuracy** |
| --- | --- | --- | --- |
| gpt-5-2025-08-07 | Basic | 0.72 (0.65-0.80) | 0.82 (0.78-0.85) |
| gpt-5-2025-08-07 | ESI-inspired | **0.77 (0.70-0.84)** | 0.82 (0.79-0.86) |
| gpt-5-mini-2025-08-07 | Basic | 0.73 (0.66-0.80) | 0.80 (0.77-0.84) |
| gpt-5-mini-2025-08-07 | ESI-inspired | **0.77 (0.70-0.84)** | 0.81 (0.78-0.85) |

**eData: The final optimized prompt provides Emergency Severity Index guidelines to the LLM.**

The final prompt is provided below, designed as a machine-readable form (XML) of the Emergency Severity Index Handbook, Fifth Edition’s flowchart ([ESI Handbook](https://media.emscimprovement.center/documents/Emergency_Severity_Index_Handbook.pdf)). The model was also provided a system prompt: “You are an Emergency Department physician.” The {}’s are replaced by patient age in years and the parsed note, respectively.

<Emergency-department-triage-rules>

Use these triage rules to guide your thinking:

Step A: Does the patient require immediate lifesaving intervention?

If yes, assign ESI 1

If no, go to Step B

Step B: Is this a high-risk situation, or does the patient have altered mental status, or severe pain/distress?

If yes, assign ESI 2

If no, go to Step C

Step C: How many resources are needed?

If none, assign ESI 5

If one, assign ESI 4

If many, go to Step D

Step D: Check vital signs (HR, RR, SpO2).

If abnormal for age or SpO2 < 92%, assign ESI 2

If not abnormal, assign ESI 3

Resources that count: labs, imaging, IV fluids, IV/IM/nebulized medications, specialty consultations, procedures.

Resources that do not count: history/physical exam, point-of-care tests, oral medications, prescription refills, phone calls, simple wound care, crutches/splints.

Pediatric fever rule:

Age under 1 month: if temperature > 38 C, assign at least ESI 2

Age 1 to 3 months: if temperature > 38 C, consider ESI 2

Age 3 months or older: if temperature > 39 C or < 36 C, or incomplete immunizations, or no clear source of fever, consider ESI 2 or 3

</Emergency-department-triage-rules>

<Prediction-task>

Below are the symptoms of two different patients presenting to the Emergency Department, Patient A and Patient B. Please return which patient is of the highest acuity between these two patients. Please return one of two answers: '0: Patient A is of higher acuity' '1: Patient B is of higher acuity' Please do not return any additional explanation.

</Prediction-task>

<Patient-A-note>

Patient A: "This patient is {} years old. {}"

</Patient-A-note>

<Patient-B-note>

Patient B: "This patient is {} years old. {}"

</Patient-B-note>

**eFigure 2: Adjusted odds ratios for incorrect LLM predictions demonstrate age-dependent trends in acuity determination.**

We assembled a single, multivariate logistic regression model to determine the odds ratio between the LLM making a mistake on a pair and the two children’s ages. A mistake means that the LLM erroneously selected the lower ESI acuity child as higher acuity. The model was adjusted for the sex, race/ethnicity, and acuity difference of both children. The table presents the odds ratios (ORs) for mistakes at different age pairings when holding all other variables constant; adolescent-adolescent pairs were selected as the reference for these age-stratified ORs. As in **Figure 1**, the LLM is generally makes fewer mistakes when the younger child is higher-acuity (light grey, top left) and makes the most mistakes when the older child is higher-acuity (dark red, bottom right). Among same-age visits (the diagonal), the LLM performs best on pairs of two adolescents.
